# Dynamics of Intersubject Brain Networks during Anxious Anticipation

**DOI:** 10.3389/fnhum.2017.00552

**Published:** 2017-11-21

**Authors:** Mahshid Najafi, Joshua Kinnison, Luiz Pessoa

**Affiliations:** Department of Psychology and Maryland Neuroimaging Center, University of Maryland, College Park, College Park, MD, United States

**Keywords:** emotion, networks, intersubject correlation, dynamics, threat, amygdala, bed nucleus of the stria terminalis (BST), periaqueductal gray (PAG)

## Abstract

How do large-scale brain networks reorganize during the waxing and waning of anxious anticipation? Here, threat was dynamically modulated during human functional MRI as two circles slowly meandered on the screen; if they touched, an unpleasant shock was delivered. We employed intersubject correlation analysis, which allowed the investigation of network-level functional connectivity across brains, and sought to determine how network connectivity changed during periods of approach (circles moving closer) and periods of retreat (circles moving apart). Analysis of positive connection weights revealed that dynamic threat altered connectivity within and between the salience, executive, and task-negative networks. For example, dynamic functional connectivity increased within the salience network during approach and decreased during retreat. The opposite pattern was found for the functional connectivity between the salience and task-negative networks: decreases during approach and increases during approach. Functional connections between subcortical regions and the salience network also changed dynamically during approach and retreat periods. Subcortical regions exhibiting such changes included the putative periaqueductal gray, putative habenula, and putative bed nucleus of the stria terminalis. Additional analysis of negative functional connections revealed dynamic changes, too. For example, negative weights within the salience network decreased during approach and increased during retreat, opposite what was found for positive weights. Together, our findings unraveled dynamic features of functional connectivity of large-scale networks and subcortical regions across participants while threat levels varied continuously, and demonstrate the potential of characterizing emotional processing at the level of dynamic networks.

## Introduction

Imagine yourself reclining on a dentist's chair. Most of us wait anxiously as the dentist gradually moves the drill toward our mouth. At the same time, if the drill is moved away (perhaps the dentist needed an additional adjustment), anxious apprehension likely will subside. A growing literature of both non-human and human research indicates that anticipatory processing of negative events engages multiple brain regions (Davis et al., 2010; Grupe and Nitschke, 2013; Tovote et al., 2015), including medial prefrontal cortex, insula, and orbitofrontal cortex, cortically. Subcortically, implicated regions include the amygdala, periaqueductal gray (PAG), and the bed nucleus of the stria terminalis (BST) (see Davis et al., 2010; Fox et al., 2015). Anticipatory negative processing allows participants to prepare and possibly minimize the impact of harmful stimuli. However, aberrant responding to uncertain future negative events is believed to be central to anxiety disorders (Grupe and Nitschke, 2013; Fox and Kalin, 2014). Thus, further elucidation of the mechanisms of anticipatory processing is important from both basic and clinical perspectives.

Anxious anticipation of negative events has widespread effects on brain function (Thomason et al., 2011; Kang et al., 2016; Raz et al., 2016; Young et al., 2017). However, understanding how the organization of large-scale brain networks is affected during anxious apprehension is poorly understood. At least three networks are altered by processing threat (Pessoa, 2013): a salience network that responds to motivationally salient stimuli (Seeley et al., 2007; Menon and Uddin, 2010); a task-negative (or “default mode”) network that is engaged when attention is directed internally and during some forms of emotional processing (Gusnard et al., 2001; Greicius et al., 2003, 2009); and an executive control network that is engaged when cognitively demanding tasks require attention (Seeley et al., 2007; Vanhaudenhuyse et al., 2011). In the context of anxious anticipation, one study described greater salience-network connectivity while participants watched an aversive movie (Hermans et al., 2011). In a previous study, we investigated network interactions when participants were in either prolonged threat (unpredictable mild shocks could be administered) or safe (no shocks possible) conditions, and characterized transient and sustained changes to the three networks above (McMenamin et al., 2014).

**Figure 1:**
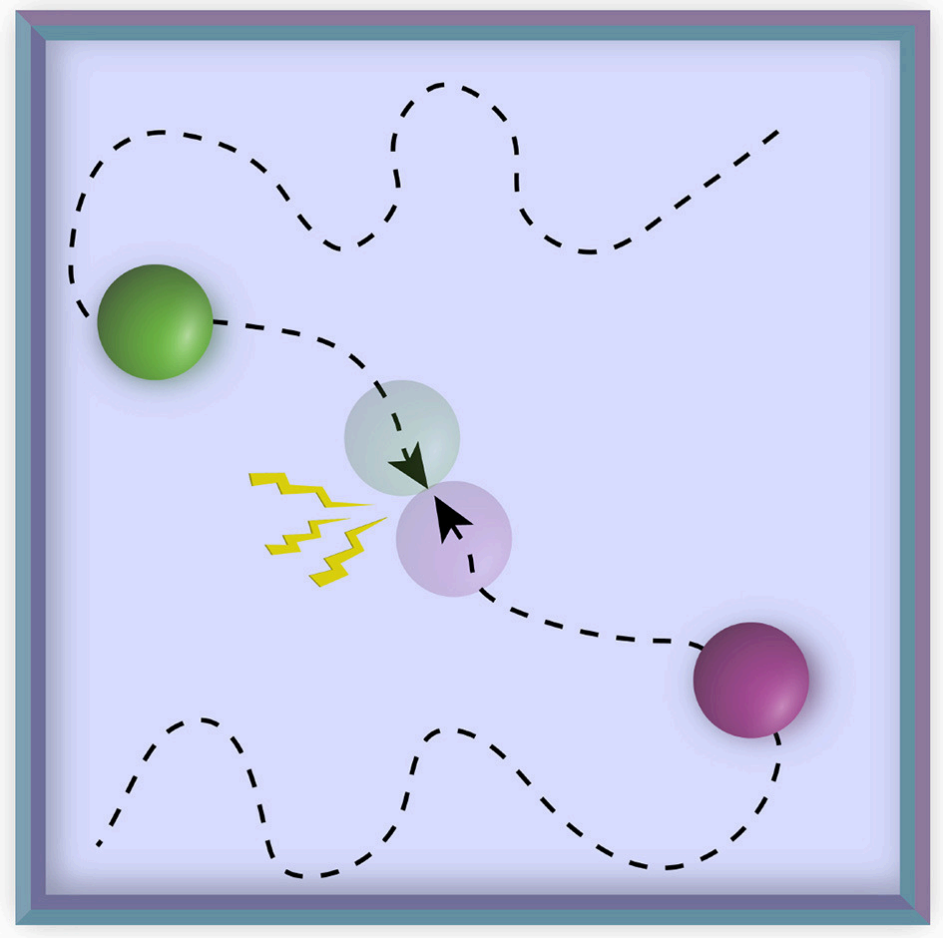
Experimental paradigm. To vary threat level dynamically, two circles moved around on the screen with some degree of randomness, sometimes approaching each other, other times retreating from each other. When they collided with each other, an unpleasant mild electric shock was delivered.

Anxious anticipation is inherently temporal. Although previous studies have investigated how brain responses are sensitive to threat proximity (Mobbs et al., 2010; Somerville et al., 2010; Grupe et al., 2013), little is known about how patterns of brain co-activation (thus networks) change during dynamic manipulations of threat. To address this gap in the literature, here we modulated threat dynamically during functional MRI scanning. Two circles moved on the screen, sometimes moving closer and sometimes moving apart (Figure 1). If they touched, an unpleasant shock was delivered to the participant. We sought to determine how functional connectivity changed during periods of approach (circles moving closer) and periods of retreat (circles moving apart). As in our previous study (McMenamin et al., 2014), we studied a set of regions spanning the salience, executive, and task-negative networks, given their involvement in cognitive and emotional processing (Yeo et al., 2011; Pessoa, 2013). In addition, we investigated subcortical regions important for emotional processing.

**Figure 2:**
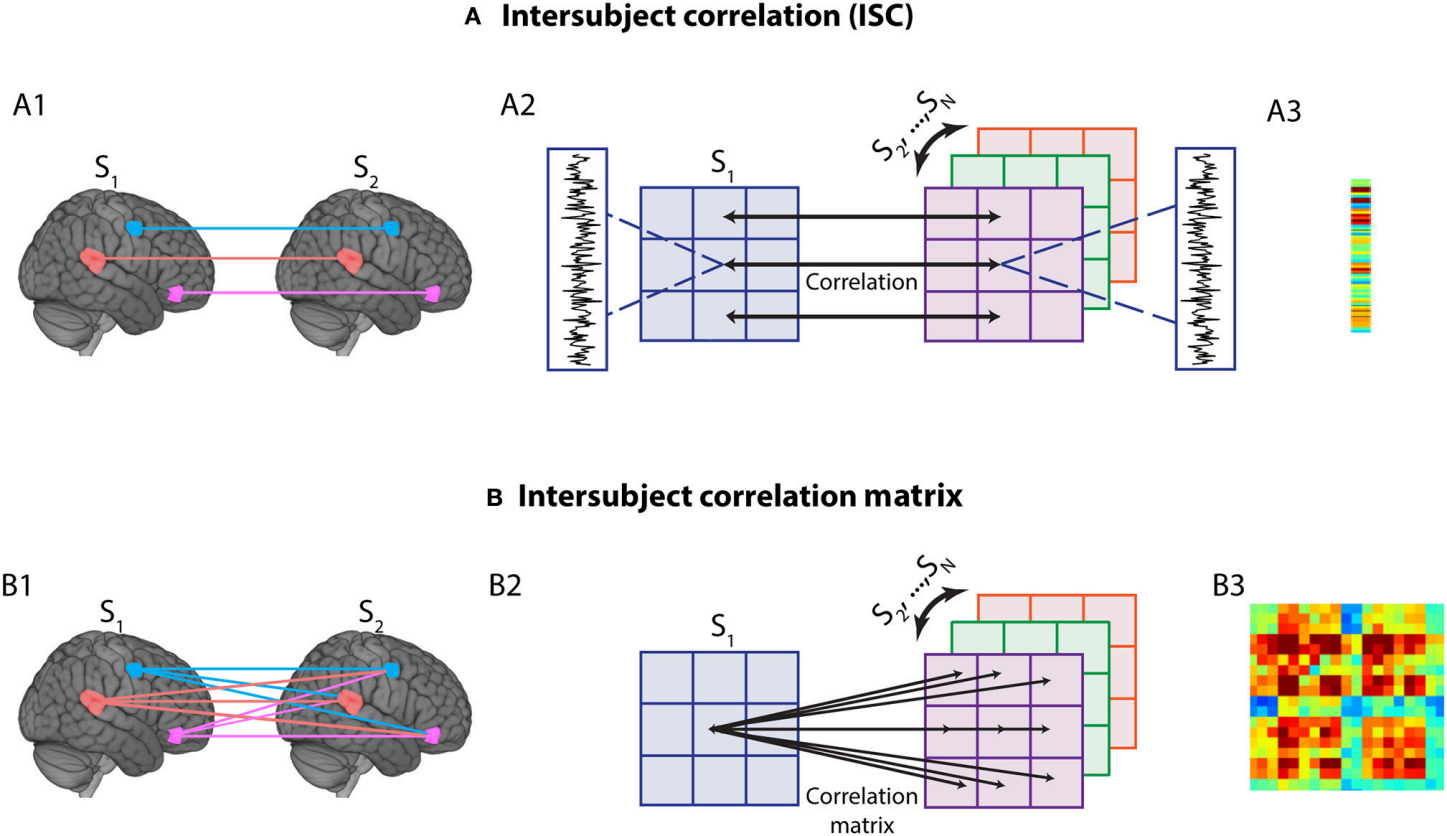
Intersubject time series analysis. **(A1)** In standard intersubject analysis, correlation between the same region across different participants' brains is calculated. **(A2)** To calculate intersubject correlation, for each voxel or region of interest (ROI), the time series for one subject (say, S1) is correlated with the average time series of all other subjects (S2,… SN), for the same voxel/ROI. This process is then iterated and averaged to determine group-level correlations, namely a vector that contains the correlation of every voxel/ROI with itself (across participants) **(A3)**. **(B1)** The method can be generalized to study multiple regions by computing the correlations between all pairs of voxels/ROIs across different brains. **(B2)** For each voxel/ROI, the time series of a one subject (say, S1) is correlated with the average time series across other subjects (S2,…,SN). This process is then iterated and averaged to determine group-level correlations, namely a matrix that contains the correlation of every voxel/ROI with every other voxel/ROI (across participants) **(B3)**. Note that the vector in A3 corresponds to the diagonal of the matrix in **B3**, illustrating that intersubject networks provide a more general characterization of time series relationships.

Overall, our approach allowed us to test several questions about the brain basis of anxious anticipation. How do functional connectivity properties of large-scale networks evolve during periods of threat approach and retreat? During dynamic threat, what is the relationship between cortical and subcortical regions important for threat processing? We investigated functional connectivity based on intersubject correlation analysis (Hasson et al., 2004), where time series data from voxels/ROIs are correlated across participants (Figure 2A). This approach can be used to compute correlations between the same region (across brains) as well as correlations between different regions (again across brains) (Figure 2B) (Najafi and Pessoa, 2016; Simony et al., 2016). Simony et al. (2016) showed that this method increased the signal-to-noise ratio in detecting functional correlations (compared to computing them within brains) likely from filtering out processing unrelated to ongoing stimulus processing, as well as non-neuronal artifacts (for example, respiratory rate, head motion) that can influence correlation patterns within a brain but are typically not correlated across brains. Another important property of intersubject network analysis is that it can consider the correlation of a region with itself; in intersubject analysis this correlation is meaningful because the time series data come from different brains.

## Materials and methods

### Participants

Ninety-three participants with normal or corrected-to-normal vision and no reported neurological or psychiatric disease were recruited from the University of Maryland community. Data from 84 participants (44 males and 40 females, ages 18–40 years; average: 22.62, STD: 4.85) were employed for data analysis (of the original sample of 93, data from 7 subjects were discarded due to technical issues during data transfer [specifically, field maps were lost], 1 subject was removed because of poor structural-functional alignment, and 1 subject's data were lost). The project was approved by the University of Maryland College Park Institutional Review Board and all participants provided written informed consent before participation. Results are reported for a group of 49 participants (23 males and 26 females, ages 18–40 years; average: 22.78, STD: 5.40). A separate group of 35 participants was used as an exploratory dataset to fix specific processing choices and to define subcortical regions of interest (ROIs).

### Procedure and stimuli

Two circles with different colors moved around on the screen randomly, and when they collided an unpleasant mild electric shock was delivered. Overall, the proximity and relative velocity of the circles were used to influence threat level. The position of each circle (on the plane), ***x***_*t*_, was defined based on its previous position, ***x***_*t*−1_, plus a random displacement, ***x***_*t*_. The magnitude and direction of the displacement was calculated by combining a normal random distribution with a momentum term to ensure motion smoothness, while at the same time remaining (relatively) unpredictable to participants. Specifically, the displacement was updated every 50 ms as follows:

$$\Delta x_{t}=(1-c)\Delta x_{t-1}+\;cN(0,1)$$

where *c* = 0.2 and *N*(0, 1) indicates the normal distribution with 0 mean and standard deviation 1. The position and amount of displacement of each circle were updated independently.

Visual stimuli were presented using PsychoPy (http://www.psychopy.org/) and viewed on a projection screen via a mirror mounted to the scanner's head coil. The total experiment included 6 runs, each of which had 6 blocks. In each block, the circles appeared on the screen and moved around for 60 s; blocks were preceded by a 15-s blank screen. Each run ended with 7 s of a blank screen.

In each of the 6 runs the circles collided a total of 8 times in 4 out of the 6 blocks (3 shocks maximum per block); each collision resulted in the delivery of an electric shock. The 500-ms electric shock was delivered by an electric stimulator (Coulbourn Instruments, PA, USA) to the fourth and fifth fingers of the non-dominant left hand via MRI-compatible electrodes. To calibrate the intensity of the shock, each participant was asked to choose his/her own stimulation level immediately prior to functional imaging, such that the stimulus would be “highly unpleasant but not painful.” After each run, participants were asked about the unpleasantness of the stimulus in order to re-calibrate shock strength, if needed.

### MRI data acquisition

Functional and structural MRI data were acquired using a 3T Siemens TRIO scanner with a 32-channel head coil. First, a high-resolution T2-weighted anatomical scan using Siemens's SPACE sequence (0.8 mm isotropic) was collected. Subsequently, we collected 457 functional EPI volumes using a multiband scanning sequence (Feinberg et al., 2010) with TR = 1.0 s, TE = 39 ms, FOV = 210 mm, and multiband factor = 6. Each volume contained 66 non-overlapping oblique slices oriented 30°Clockwise relative to the AC-PC axis (2.2 mm isotropic). In addition, a high-resolution T1-weighted MPRAGE anatomical scan (0.8 mm isotropic) was collected. Finally, double-echo field maps (TE1 = 4.92 ms, TE2 = 7.38 ms) were acquired with acquisition parameters matched to the functional data.

### Exploratory and test data sets

Data from 84 participants were employed in this study. One of our goals was to attempt to enhance reproducibility in a research area that faces the “curse of flexibility.” For example, a recent review enumerated 69,120 different workflows for basic functional MRI analysis alone (Poldrack et al., 2017). We thus employed an exploratory data set to fix specific processing choices, and to define subcortical ROIs, as described in this section. At a point during data collection when approximately 40 participants had been studied, we labeled the data as “exploratory” and to be used for data exploration. A total of *N* = 35 usable participants were used in the exploratory set. With the entire processing pipeline and ROIs fixed, statistical testing was then applied to a separate dataset (*N* = 49; the original goal being to have approximately 50 participants).

### Stimulus conditions

The exploratory set was used to define two conditions, “approach” and “retreat,” based on whether the circles were moving toward or away from each other. Time points were only considered for analysis if the Euclidian distance between the circles was at least 75% of the maximum distance that the circles exhibited during the whole experiment; otherwise, the data were not employed in the analysis. The rationale behind this was that, when the circles were far from each other, participants reported that they did not really pay as much attention to them. Therefore, we reasoned that the analysis should focus on the time points during which the circles were in (relative) closer proximity to each other. Investigation of the exploratory set revealed that the particular cutoff was not critical for the effects investigated and that values at least between 65 and 85% were adequate; we chose a cutoff value of 75%. After applying the cutoff, the approach condition included 481 data points (i.e., TRs) and the retreat condition 290 data points.

### Functional MRI preprocessing

We employed the exploratory data set to define the following preprocessing steps. Skull stripping determines which voxels are to be considered part of the brain and, although conceptually simple, plays a very important role in successful subsequent co-registration and normalization steps. Currently, available packages perform sub-optimally in specific cases, and mistakes in the brain-to-skull segmentation can be easily identified. Accordingly, to skull strip the T1 high-resolution anatomical image (which was rotated to match the oblique plane of the functional data with AFNI's 3dWarp), we employed six different packages: ANTs (Avants et al., 2011), AFNI (Cox, 1996; http://afni.nimh.nih.gov/), ROBEX (Iglesias et al., 2011; https://www.nitrc.org/projects/robex), FSL (http://fsl.fmrib.ox.ac.uk/fsl/fslwiki), SPM (http://www.fil.ion.ucl.ac.uk/spm), and Brainsuite (Shattuck and Leahy, 2002). We employed a “voting scheme” as follows: based on T1 data, a voxel was considered to be part of the brain if 4/6 packages estimated it to be a brain voxel; otherwise the voxel was not considered to be brain tissue (for 6 subjects whose T1 data were lost due to issues during data transfer, the T2 image was used instead and only the ANTs package was used for skull-stripping).

Subsequently, FSL was used to process field map images and create a phase-distortion map for each participant (with bet and fsl_prepare_fieldmap). FSL's epi_reg was then used to apply boundary-based co-registration to align the unwarped mean volume registered EPI images with the skull-stripped anatomical image (T1 or T2) along with simultaneous EPI distortion-correction (Greve and Fischl, 2009). Next, ANTS was used to determine a non-linear transformation that mapped the skull-stripped anatomical image (T1 or T2) to the MNI152 template (interpolated to 1-mm isotropic voxels). Finally, ANTs combined the non-linear transformations from co-registration/unwarping (from mapping mean functional EPI images to the anatomical T1 or T2) and normalization (from mapping T1 or T2 to the MNI template) into a single transformation that was applied to map registered functional volumes of functional data to standard space (interpolated to 2-mm isotropic voxels). In this process, ANTs also utilized the field maps to simultaneously minimize EPI distortion.

Additional preprocessing steps included the following. The first three volumes of each functional run were discarded to account for equilibration effects. Slice-timing correction (with AFNI's 3dTshift) used Fourier interpolation to align the onset times of every slice in a volume to the first acquisition slice, and then a six-parameter rigid body transformation (with AFNI's 3dvolreg) corrected head motion within and between runs by spatially registering each volume to the first volume.

### Subcortical ROIs

We used the exploratory data set to investigate subcortical ROIs focusing on functional to anatomical co-registration. The subcortical ROIs selected for evaluation in the test set included the amygdala, PAG, habenula, and BST. For the amygdala, we considered two subregions defined in the Nacewicz et al. (2014) parcellation, specifically: lateral amygdala and central/medial amygdala. For the PAG, we modified the mask by Roy et al. (2014), which was dilated by 1 voxel; in addition, we manually removed voxels overlapping cerebrospinal fluid. The habenula has been implicated in emotional/motivational processing (Hikosaka, 2010; Mizumori and Baker, 2017), and here we employed a mask defined according to the Morel atlas, as defined in Krauth et al. (2010). For the BST, we employed a recently developed mask based on 7 Tesla data but defined having in mind 3 Tesla acquisition (Theiss et al., 2017).

### ROIs for large-scale networks

We investigated three networks widely studied in the literature: salience, executive, and task negative. From these networks, we employed cortical ROIs (defined as 5-mm radius spheres) based on the center coordinates provided by previous studies (Table 1): salience network (Hermans et al., 2011) (13 regions), executive network (Seeley et al., 2007) (12 regions), and task-negative network (Fox et al., 2005) (12 regions). If two ROIs abutted each other, each mask was eroded by 1 voxel from the touching boundary to minimize any potential data “spill over.”

**Table 1:** List of cortical and subcortical Regions of Interest (ROIs).

**ROI names**	**Coordinates (MNI)**		
	***x***	***y***	***z***
**SALIENCE**			
1) Frontoinsular cortex L	−34	18	4
2) Frontoinsular cortex R	34	22	4
3) Dorsal anterior cingulate cortex	2	10	40
4) Temporo-parietal junction L	−62	−26	36
5) Temporo-parietal junction R	62	−26	36
6) Inferotemporal cortex L	−54	−62	−4
7) Inferotemporal cortex R	54	−54	−8
8) Precentral L	−26	−6	64
9) Precentral R	26	−2	64
10) Dorsolateral prefrontal cortex L	−38	42	24
11) Dorsolateral prefrontal cortex R	34	46	28
12) Inferior frontal gyrus L	−54	6	20
13) Inferior frontal gyrus R	54	10	12
**EXECUTIVE**			
14) Orbital frontoinsula L	−36	24	−10
15) Dorsolateral prefrontal cortex R	46	46	14
16) Dorsolateral prefrontal cortex L	−34	46	6
17) Ventrolateral prefrontal cortex R	34	56	−6
18) Ventrolateral prefrontal cortex L	−32	54	−4
19) Frontal operculum R	56	14	14
20) Dorsolateral prefrontal cortex / frontal eye field R	30	12	60
21) Dorsolateral prefrontal cortex / frontal eye field L	−32	18	50
22) Dorsomedial prefrontal cortex	0	36	46
23) Lateral parietal R	38	−56	44
24) Lateral parietal L	−48	−48	48
25) Inferior temporal R	58	−54	−16
**TASK NEGATIVE**			
26) Posterior cingulateprecuneus (PCC)	−3	−38	38
27) Retro-splenial	2	−52	9
28) Lateral parietal cortex (LP) L	−50	−64	38
29) Lateral parietal cortex (LP) R	50	−64	38
30) Medial prefrontal cortex (MPF) L	−4	42	−9
31) Medial prefrontal cortex (MPF) R	0	59	16
32) Superior frontal L	−16	44	51
33) Superior frontal R	17	43	51
34) Inferior temporal L	−62	−33	−20
35) Inferior temporal R	66	−18	−20
36) Parahippocampal gyrus L	−22	−26	−20
37) Parahippocampal gyrus R	25	−26	−18
**SUBCORTICAL REGIONS**			
38) Amygdala Central-Medial L			
39) Amygdala Central-Medial R			
40) Amygdala Lateral L			
41) Amygdala Lateral R			
42) Periaqueductal gray (PAG) L			
43) Periaqueductal gray (PAG) R			
44) Habenula L			
45) Habenula R			
46) Bed nucleus of the stria terminalis (BST) L			
47) Bed nucleus of the stria terminalis (BST) R			

*Cortical ROIs were defined via 5-mm radius spheres centered on the MNI coordinates provided below. Subcortical ROIs were defined anatomically (see text for details)*.

### Intersubject correlation analysis

We investigated functional connections based on intersubject correlation analysis (Hasson et al., 2004). This was the first step from which temporal dynamics was evaluated (see below).

**Figure 3:**
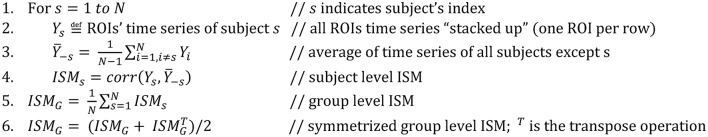
Computation of group-level intersubject correlation matrix (ISM). The operator **corr** corresponds to Pearson correlation.

To perform intersubject correlation analysis, time series data from ROIs were correlated across participants, that is, across different brains (Figure 2A). A simple, yet powerful extension to intersubject correlation analysis is to consider intersubject correlations across all pairs of ROIs, which allows the application of the technique to networks (Figure 2B) (Najafi and Pessoa, 2016; Simony et al., 2016). The procedure to generate an intersubject network is as follows (Figure 3). For a given ROI, one subject's s data are held out (***y***_*s*_), and the rest of the subjects' time series is averaged (***y***_−*s*_). Then, the Pearson correlation between these two data vectors is computed: corr(***y***_*s*_, ***y***_−*s*_). This basic operation is repeated for all pairs of ROIs to compute an intersubject matrix for the held-out subject (ISM_S_). Thus, the ISM_S_ is an *NxN* matrix, where *N* is the number of ROIs, and the *ij*-th matrix element contains the correlation coefficient between the *i*-th ROI time series of the held-out subject and the *j*-th ROI time series averaged across the remaining subjects.

This procedure is repeated for all subjects. A group matrix (ISM_G_) is then obtained by averaging across all subjects; the distribution of intersubject correlations was well approximated by a Gaussian (mean: −0.0022; STD: 0.0181) so averaging them is reasonable. Note that the resulting intersubject network is not necessarily symmetric, because, for each ROI, the time series in the held-out subject (***y***_*s*_) is not necessarily equal to average of all other subjects' time series (***y***_−*s*_) (symmetry is desirable because the intersubject correlation between regions *i* and *j* should, in principle, be equal to the intersubject correlation between regions *j* and *i)*. While the ISM_G_ matrix in practice will be near-symmetric, a simple and intuitive way to mathematically accomplish symmetry is to average the group-level intersubject network with its transpose (where row and column indexes are flipped), leading to a final symmetric group matrix. Finally, the procedures above were performed, separately, for the approach and retreat conditions (generating one matrix per condition). Note that in our method the matrix diagonal is also computed because data at a given diagonal entry [*i, i*] is computed across different brains.

### Time series data

Because intersubject analysis seeks to investigate correlations across brains of different individuals, the contributions of the paradigm were not regressed out as done in some within-subject analyses. However, because the moving circles were presented in 60-s blocks that alternated with 15-s rest period, we regressed out (with AFNI's 3dDeconvolve) the block effect (which was convolved with the standard hemodynamic response; (see Cohen, 1997). Other regressors in the model included 6 motion parameters (3 linear displacements and 3 angular rotations), and their discrete temporal derivatives. Additionally, to model baseline and drifts of the MR signal, regressors were included corresponding to polynomial terms up to 4th order (for each run separately). To minimize the shock effect, data points in a 15-s window after shock delivery were discarded from all analyses. In addition, to minimize the impact of potential block onsets (which can elicit strong transients), for each block, the first 15 time points (15 s) were discarded. Finally, the residual time series for each run was z-scored separately.

The residual time series as defined was used for the intersubject correlation analysis. As approach and retreat varied dynamically throughout the blocks, we employed a windowing procedure to extract data segments corresponding to approach and retreat periods. Intuitively, the windowing allowed us to select segments of the time series associated with each condition and concatenate them across all runs, as outlined next.

### Dynamic intersubject correlation

**Figure 4:**
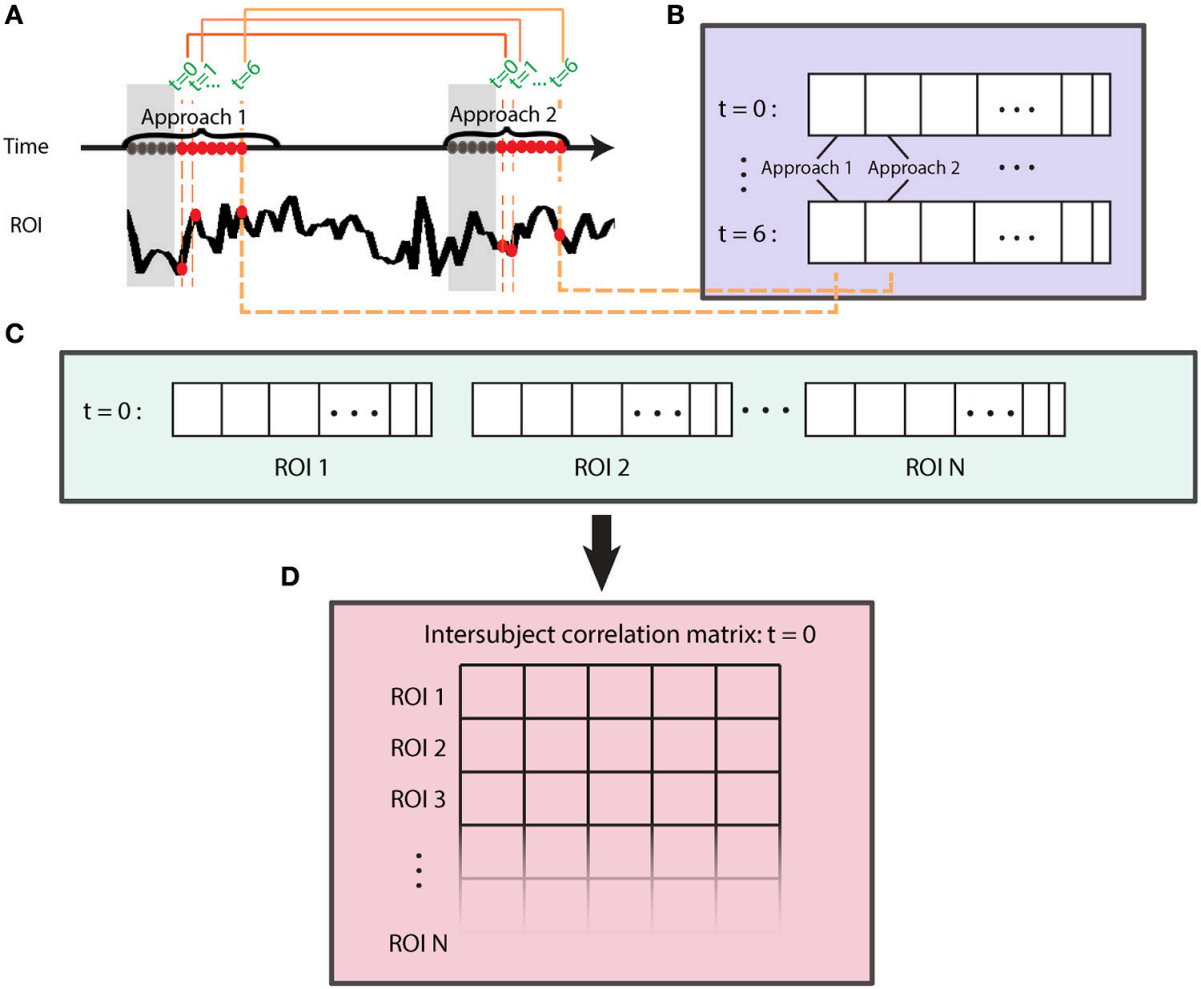
Evaluation of intersubject functional connectivity dynamics. Intersubject correlation matrices were computed at each time point of approach and retreat segments, separately. **(A)** Time was used to index data during approach and retreat (not shown) segments. To account for the hemodynamic delay, we discarded the first 5 s of each segment (gray part). **(B)** A time series vector at *t* = 0 was generated by concatenating all of the *t* = 0 samples (across same-condition segments across blocks and runs); likewise, for *t* = 1, and so on. **(C)** At a given time *t*, time series vectors were used to compute the intersubject correlation matrix **(D)**. This process is shown for *t* = 0 and repeated for times *t* = 1,…,6 for approach and *t* = 1, …5 for retreat.

To investigate dynamic aspects of functional connectivity, we first computed intersubject correlations as indicated previously (see Figure 3) at each time *t*. The method considered all approach and retreat segments separately, and computed one vector for each time *t*, ROI, and condition (Figure 4). Specifically, for each segment type (approach and retreat, separately), we considered all time series data at *t* = 0 time points, then *t* = 1 time points, and so on, separately (Figure 4A). The goal was to generate a vector of data at *t* = 0 by concatenating all of the *t* = 0 data across approach/retreat segments. To do so, we concatenated the *t* = *k* points (for a fixed *k*) across segments (Figure 4B). To account for the hemodynamic delay, we discarded the first 5 s of each segment. The overall process allowed us to compute correlations across subjects for ROI pairs each time t (Figure 4C), thus computing the matrices ISM_t = 0_, ISM_t = 1_, etc. (Figure 4D). We considered intersubject matrices for *t* = 0, …, 6 s for the approach condition and *t* = 0, …, 5 s for the retreat condition. For both conditions, at least 20 data points (Figure 4B) were available per condition and time slice (but note that less data were available for the retreat compared to the approach condition, as some points were discarded following shock events). Whereas, longer periods of approach/retreat occurred, they did not occur at least 20 times, which was the minimum number of repetitions that we judged needed for stable assessment of the correlations. Given this constraint, there were 443 data points for approach and 255 for retreat.

### Within- and between-network weight

To measure the strength of connections within and between networks, we utilized within- and between-network connectivity weights, respectively. The weight between network *N*_1_ and network *N*_2_, *W*_*N*_1_, *N*_2__, was defined as follows:

(1) $$W_{N_{1},N_{2}}=\frac{1}{n_{1}n_{2}}\sum_{i\in N_{1},j\in N_{2}}c_{ij}$$

where *n*_*i*_ is the number of ROIs in network *N*_*i*_. The value *c*_*ij*_ is the ISM_G_ value when the *i*-th ROI belongs to network *N*_1_ and the *j*-th ROI belongs to network *N*_2_. When *N*_1_ and *N*_2_ are the same network, the formula calculates within-network weight. For most of the analyses reported here, we considered only positive weights, as commonly done in network research (but see below for negative weights). Most network measures do not handle self-connections (Newman, 2010), which in standard analysis (when correlations are determined within participants) are not informative (*c*_*ii*_ = 1). Here, we considered functional connections between the same region (across participants), which could be incorporated in within-network weight by considering the terms *c*_*ii*_ in Equation (1).

To study dynamic changes to network cohesion, ISM_G_ was computed as outlined above at each time *t* and within- and between-network weights computed. Linear regression was used to evaluate changes in network weight during approach and retreat conditions. To evaluate functional connections between subcortical regions and the salience network, we computed a weight index that summed all functional connections between a specific subcortical region and all nodes of the salience network. This was performed for the approach and retreat conditions, separately.

Because intersubject correlations are not independent (each participant is present in more than one participant pair), statistical tests were performed via non-parametric resampling tests based on the approach proposed by Kauppi et al. (2014). Briefly, we computed a null resampling distribution by circularly shifting each participants' time-series by a random amount so that they were no longer aligned in time across participants, and then calculated intersubject correlations. The null distribution was estimated by computing 100,000 realizations (fewer iterations produced very similar results showing that the estimation was stable). Statistically, we were interested in evaluating changes to network weight during approach and retreat, separately, and comparing the slopes of the linear fits during approach vs. retreat. Thus, observed slopes (based on data) were compared to values of the corresponding null distribution and *p*-values determined; for the contrast of approach vs. retreat, the observed difference (based on data) was compared to values of the corresponding difference null distribution.

**Figure 5:**
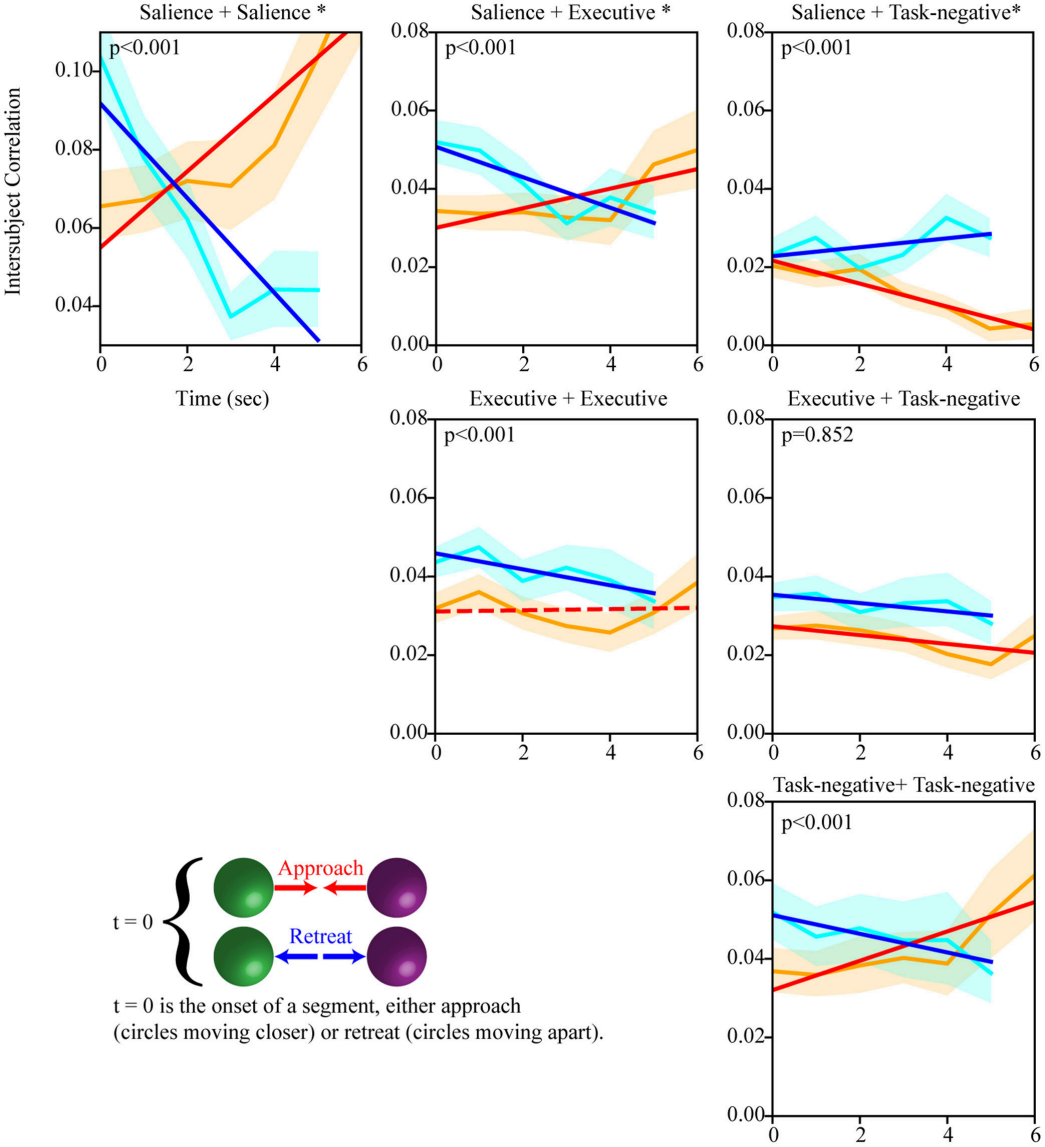
Temporal evolution of intersubject network weight during approach and retreat segments (positive weights). Within- and between-network weights during approach and retreat for the salience, executive, and task-negative networks. For example, as the circles approached each other, the weights within the salience network increased; when the circles retreated, within-network weights decreased. Slightly longer approach periods were available to compute dynamics for this condition. The orange line shows approach weights (90% confidence band); the cyan line shows retreat weights (90% confidence band). The red/blue line show the least-squares linear fit to weight values during approach/retreat; solid lines indicate fits such that *p* < 0.05. Time is in seconds; the y-axis shows within/between weight. The asterisk indicates that the slope difference was such that *p* < 0.05.

We observed that the mean of the null distributions for approach and retreat were not zero but small positive values (which were due to the unequal number of time points used for segments of different duration; for example, there were fewer segments of duration 5 s than 4 s). Thus, the mean values expected by chance (determined via the null distribution) were subtracted from the observed values (Figures 5, 7).

To explore dynamic changes in negative weights, we considered equation (1) by excluding all positive weights and followed the same procedures described above for positive weights. The null distribution was based on 100,000 realizations.

## Results

We performed intersubject correlation analysis during threat approach and retreat. We focused on regions of the salience, executive, and task-negative networks, as well as a targeted set of subcortical ROIs. As threat level was varied dynamically, we investigated how the intersubject correlation matrix evolved temporally. Figure 5 shows the temporal evolution of within- and between-network weight during approach and retreat for the three networks. Statistically, we evaluated changes during approach and retreat via linear regression, as well as the difference between the two; for ease of reference, statistical values are provided in the figure (slopes at *p* < 0.05 are indicated by solid lines, and *p*-values for the difference in slopes are provided).

**Figure 6:**
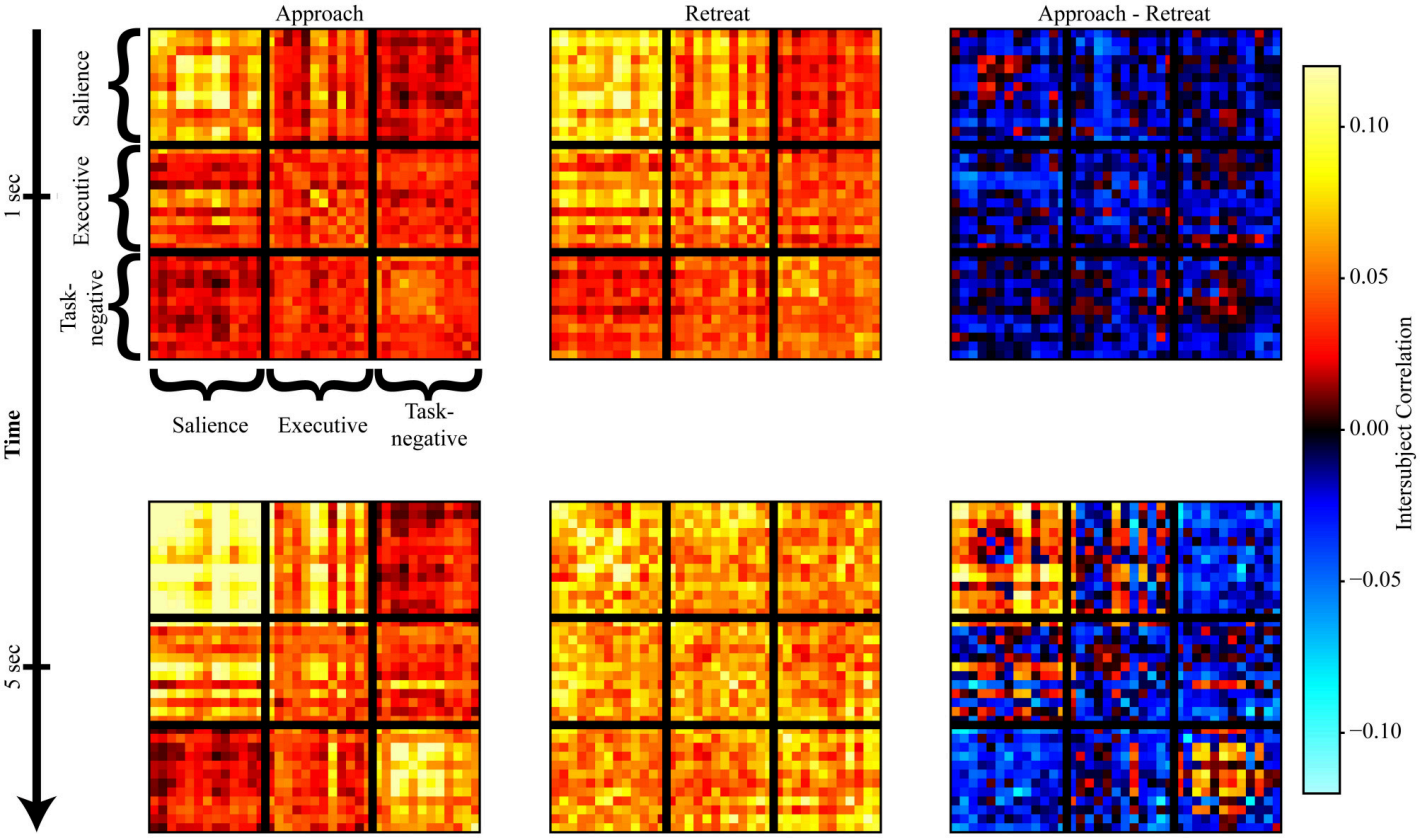
Snapshots of intersubject correlation matrices. Results are shown for approach and retreat conditions, as well as approach minus retreat. The top row shows intersubject correlations at *t* = 1 s, and the bottom row shows intersubject correlations at *t* = 5 s (in both cases, a hemodynamic lag of 5 s was employed).

Evaluation of within-network weight revealed that all networks exhibited functional connectivity changes during approach and/or retreat periods, with the strongest change observed for the salience network. During retreat, weight decreased within all networks. Changes in intersubject correlation were also found between networks; specifically, between the salience and executive networks, and between the salience and task-negative networks. The weight between the salience and executive networks increased during approach and decreased during retreat. However, the opposite was found between the salience and task-negative networks. In this case, the correlation actually decreased during approach. For illustration purposes only, Figure 6 shows changes of the group-averaged correlation matrix of the three large-scale networks (negative values were removed from each participant's matrix).

**Figure 7:**
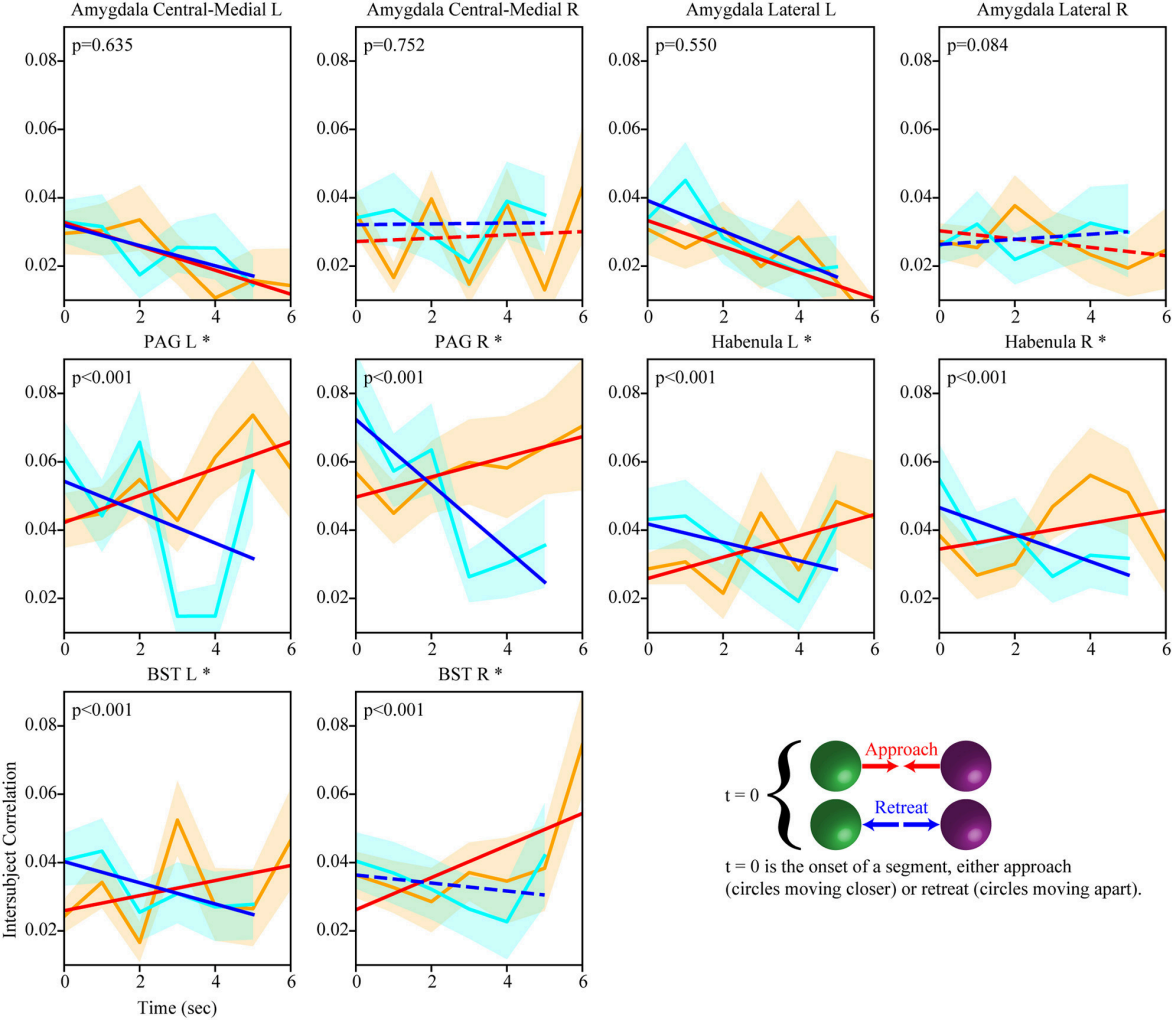
Temporal evolution of intersubject weights between subcortical regions and the salience network (positive weights). Dynamic changes in intersubject correlation between subcortical regions and the salience network were detected for both approach and retreat. For example, for the right PAG, intersubject correlation increased during approach and decreased during retreat. Conventions as in Figure 5.

**Figure 8:**
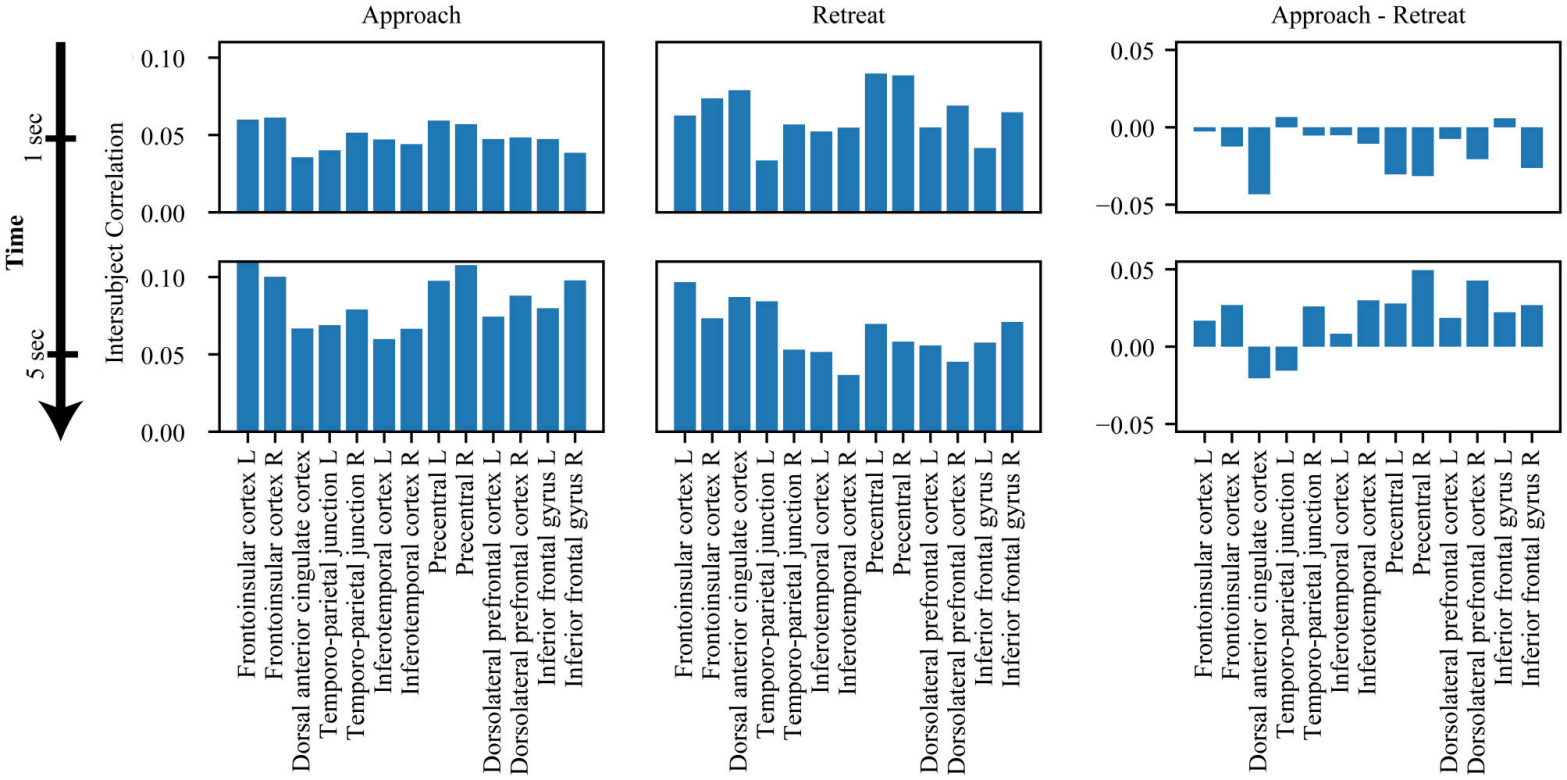
Snapshots of intersubject correlations between right PAG and salience network. Results are shown for approach and retreat conditions, as well as approach minus retreat. The top row shows intersubject correlations at *t* = 1 s, and the bottom row shows intersubject correlations at *t* = 5 s. Intersubject correlations between the right PAG and the salience network increased during approach and decreased during retreat.

We also investigated the evolution of the functional interactions between targeted subcortical regions and the salience network. In most cases, their correlations with the salience network were dynamic (Figure 7). The PAG, habenula, and BST exhibited similar patterns of dynamic intersubject correlations with the salience network, which increased during approach and decreased during retreat (for the right BST, changes during retreat were not detected). Figure 8 illustrates changes between the right PAG and the entire set of salience network ROIs. For the amygdala regions, when changes were detected, only decreases in correlation were observed during both approach and retreat segments, and the slopes were comparable in both conditions.

### Negative weights

The results above did not consider negative weights. Whereas, the step of thresholding negative weights is commonly adopted in analysis of brain networks, investigation of changes in negative weights is likely to be informative. For example, negative correlations between two systems might indicate that they are complementary or that they work in some form of opposition (such as appetitive and aversive systems). More generally, effective ways of handling negative ways remain an open question (Rubinov and Sporns, 2011; Fornito et al., 2016), as positive and negative weights may index qualitatively different processes. Possible approaches include considering the absolute value of connections (thus disregarding polarity), adding a constant positive value to all weights (effectively making all weights positive and negative weights smaller than positive ones), or in the present context summing positive and negative connections (which could lead to positive and negative weights “canceling out”).

**Figure 9:**
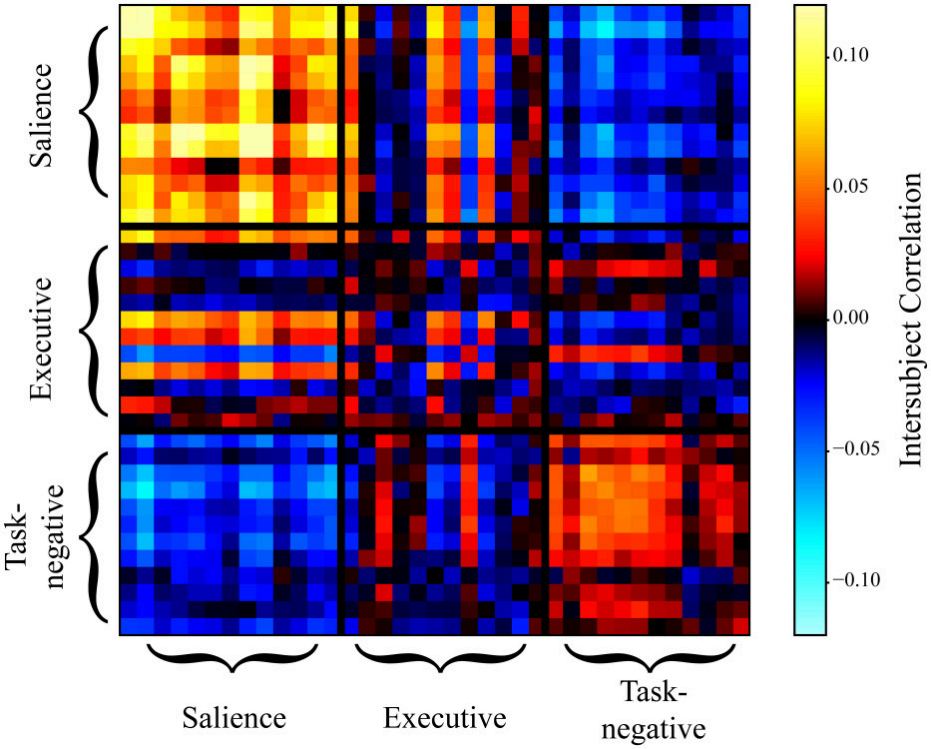
Intersubject correlation matrix during approach when both positive and negative weights were simultaneously considered (no thresholding). Weights between the salience and task-negative networks were predominantly negative, indicating that they were inversely related during approach. For simplicity, all data points during individual approach segments were averaged; likewise, during individual retreat segments.

**Figure 10:**
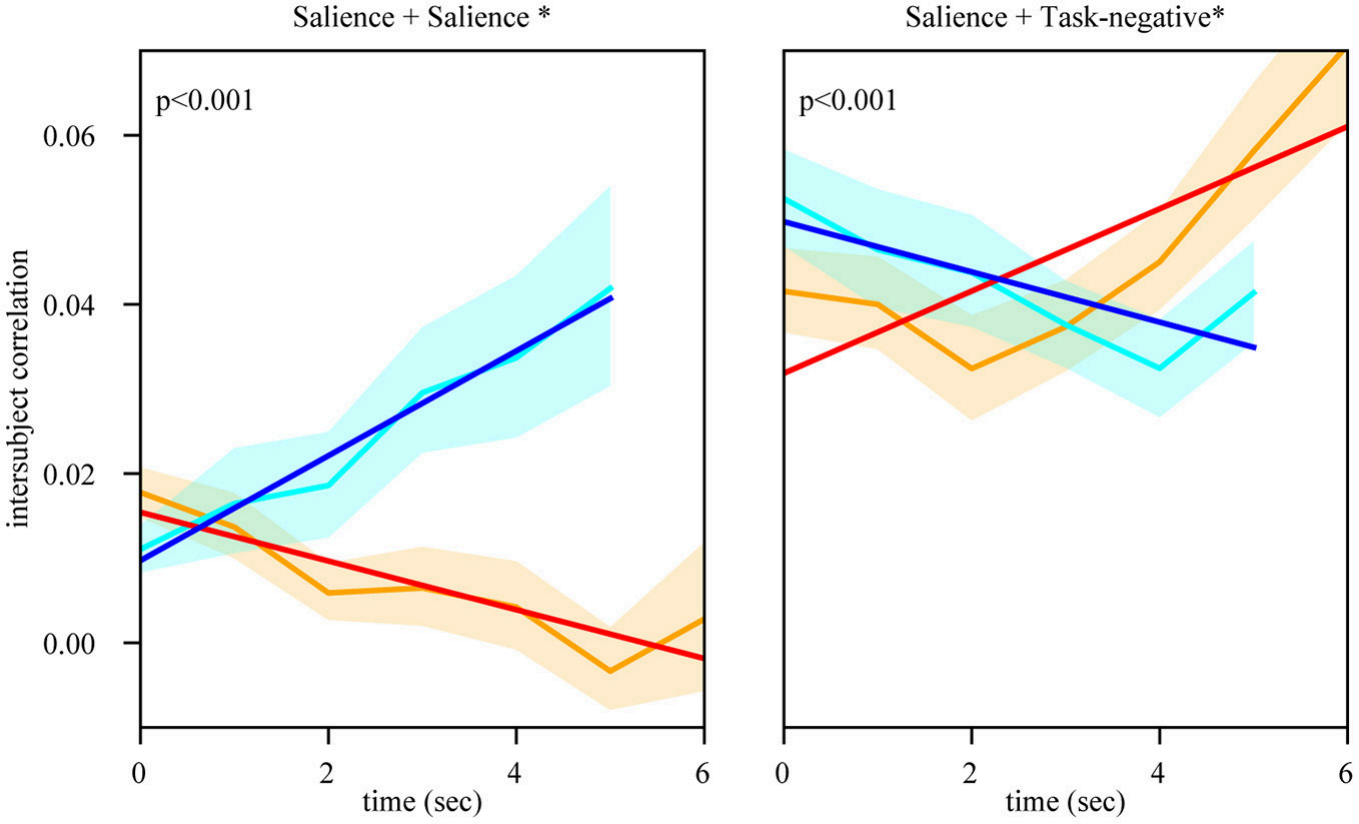
Temporal evolution of intersubject negative weights during approach and retreat segments. Within the salience network, negative weights increased during retreat and decreased during approach. Between the salience and task-negative networks, negative weights increased during approach and decreased during retreat. In both cases, the dynamic pattern of negative functional connectivity was the opposite of what was observed for positive weights (compare with Figure 5). Conventions as in Figure 5.

Inspection of the intersubject correlation matrix without any thresholding (that is, considering positive and negative weights in equation 1) revealed a predominance of negative weights between the salience and task-negative networks (Figure 9). To explore potential information of negative weights, we considered equation (1) but only for negative weights (thus, positive weights were excluded). We focused on the weights within the salience network and between the salience and task-negative networks (Figure 10). Salience-network negative weights increased during retreat and decreased during approach. Negative weights between the salience and task-negative networks increased during approach and decreased during retreat. These results were thus opposite to what was observed for positive weights.

## Discussion

In the present study, we employed intersubject functional correlation analysis to investigate large-scale networks during threat approach and retreat. We found that functional connectivity within and between networks changed dynamically as threat imminence increased and decreased (as circles moved closer and farther to/from each other).

Standard intersubject correlation analysis has been used to investigate “synchrony” across brains when participants watch the same movie or during other naturalistic conditions, such as hearing extended narratives (Hasson et al., 2004; Stephens et al., 2010; Nummenmaa et al., 2012, 2014). The method was recently extended so that a specific voxel/region in one person could be correlated with multiple voxels/regions in other participants (Najafi and Pessoa, 2016; Simony et al., 2016). Our interpretation of intersubject correlation is less tied to inter-personal synchronization (possibly tied to social processes), but more to the method's potential at reducing contributions of processing unrelated to the task/conditions at hand. Furthermore, intersubject analysis increases the signal-to-noise ratio by filtering out unwanted contributions to the measured BOLD signal (Simony et al., 2016). This is particularly important for head motion, which can induce within-participant correlations (Van Dijk et al., 2012). By computing correlations across participants, the approach essentially eliminates this issue if head motion is uncorrelated across participants (here, head motion parameters exhibited a correlation across subjects of 0.02 in the test set).

A central finding of our study was that functional connectivity within and between networks changed dynamically during periods of approach and retreat. Thus, networks are not static but dynamic entities (Bassett et al., 2011; Pessoa and McMenamin, 2016; Pessoa, 2017). This adds to findings from recent studies that showed how large-scale networks are reorganized during periods of threat (Hermans et al., 2011; McMenamin et al., 2014). The positive functional connections within the salience network increased during approach, and decreased during retreat. Thus, the salience network became more cohesive (regions more correlated) during approach in line with previous studies (Hermans et al., 2011; McMenamin et al., 2014), and less cohesive during retreat. In contrast, the correlation between the salience and task-negative networks increased during retreat, but decreased during approach. Overall, our findings demonstrate that network functional connectivity is a dynamic property that depends on threat level.

We also explored the evolution of negative weights. Negative functional connections within the salience network followed the opposite pattern to that observed for positive weights. These results are also consistent with the notion that the salience network becomes more cohesive during approach, as the negative weights between nodes of the network decreased during this period (the increase of negative weights during retreat suggests that there is was a tendency for anti-correlation to also occur). The negative weights between the salience and task-negative networks also displayed a pattern opposite that found for positive functional connections. This result was noteworthy because studies have reported that the salience and task-negative networks are anti-correlated (Uddin et al., 2009). Here, such negative correlation was found to dynamically increase during approach periods (and to decrease during retreat).

The salience network comprises multiple regions in parietal, frontal, and insular cortices (Seeley et al., 2007; Menon and Uddin, 2010). Sometimes subcortical regions are listed as part of the network, most notably the amygdala and PAG (Seeley et al., 2007); the latter is an important brain region involved in threat processing (Bandler and Shipley, 1994). In the present study, not only were multiple subcortical regions functionally connected with the salience network, but the correlations evolved during periods of approach and retreat. Of the subcortical regions investigated, the PAG, habenula, and BST exhibited a pattern of dynamic connectivity that resembled most clearly that of the salience network itself, namely increasing connectivity during approach and decreasing connectivity during retreat.

Whereas the amygdala is engaged by emotion-laden stimuli and conditions involving acute threat (as in aversive conditioning paradigms), its involvement in potential threat (where threat is not proximal and is relatively uncertain) is less clear (Davis et al., 2010). Some human neuroimaging studies have even observed deactivations of the amygdala during conditions of potential threat (Pruessner et al., 2008; Wager et al., 2009; Choi et al., 2012). Here, we observed decreases in correlation with the salience network during both approach and retreat in amygdala subregions (but only on the left hemisphere). Perhaps the most noteworthy aspect of these results is that they clearly followed a different pattern compared to the PAG, habenula, and BST.

The involvement of the BST in potential threat was suggested in early work by Davis and colleagues (Davis and Shi, 1999) and has been investigated recently in rodent studies with new neurotechnologies (see Tovote et al., 2015). Work in humans has revealed the involvement of the BST in potential threat, too (for reviews, see Fox et al., 2015; Shackman and Fox, 2016). The BST is rather small and thus challenging to investigate in humans with functional MRI. Nevertheless, recent work at higher resolution and magnetic field strengths (such as 7 Tesla) has been used to generate anatomical masks (Avery et al., 2014; Torrisi et al., 2015), and these appear to be reasonable approximations even at the standard field strength of 3 Tesla (Theiss et al., 2017). An open question concerns the conditions leading to BST engagement. While some studies suggest that uncertainty may be a major determinant of BST responses (Alvarez et al., 2011), this is not entirely clear. For example, a previous study reported greater BST responses for a simple threat approach vs. retreat contrast (the authors only employed a single level of approach vs. retreat “level;” also, the activation pattern was very diffuse, thus hard to attribute to the BST with more confidence) (Mobbs et al., 2010). In the present study, functional connectivity between the BST and the salience network increased during approach; decreased connectivity was only detected in the left BST.

Although the involvement of the habenula in affective/motivational processes has been known for some time (Butler and Hodos, 2005), there is renewed interest in the function of this structure (Hikosaka, 2010; Mizumori and Baker, 2017). The habenula is a small structure (located at the posterior-dorsal-medial end of the thalamus) and we must exert caution in attributing our results to this structure without further confirmation. Nevertheless, the putative habenula increased functional connectivity to the salience network during approach, and decreased connectivity during retreat. In this context, we reiterate that we investigated functional connectivity of several regions that are challenging to image with functional MRI, including amygdala subnuclei, PAG, and BST (in addition to the habenula). Although great care was taken at co-registration and functional data were not smoothed, we suggest that region labels be considered “putative” insofar as higher functional resolution would be required for clearer anatomical attribution.

In conclusion, we employed intersubject functional correlation analysis, which allows the investigation of functional connectivity “across brains.” We detected dynamic changes in functional connectivity involving regions across the salience, executive, and task-negative networks, as well as subcortical regions. Importantly, functional connectivity within and between networks changed dynamically as threat imminence increased and decreased. For example, positive dynamic functional connectivity increased within the salience network during approach and decreased during retreat. Functional connections between several subcortical regions and the salience network also changed dynamically during approach and retreat periods. The regions included the PAG, habenula, and BST. Taken together, our findings unraveled dynamic features of large-scale networks while threat levels varied continuously. The results demonstrate the potential of characterizing dynamic emotional processing at the level of large-scale networks, and not simply at the level of evoked responses in specific brain regions.

## Author contributions

MN and LP designed research; MN, JK, and LP analyzed the data; LP and MN wrote the paper.

### Conflict of interest statement

The authors declare that the research was conducted in the absence of any commercial or financial relationships that could be construed as a potential conflict of interest. The reviewer ASH and handling Editor declared their shared affiliation.
